# The relationship between the flexor and extensor muscle areas and the presence and degree of intervertebral disc degeneration in the cervical region

**DOI:** 10.1097/MD.0000000000031132

**Published:** 2022-10-21

**Authors:** Yavuz Yuksel, Tarkan Ergun, Ebru Torun

**Affiliations:** a Department of Radiology, Faculty of Medicine, Alaaddin Keykubat University, Alanya, Turkey.

**Keywords:** cervical, intervertebral disc degeneration, magnetic resonance imaging, spine

## Abstract

This study aimed to investigate the relationship between the presence and degree of cervical intervertebral disc degeneration (IVDD) and the cervical region muscle areas.

**Methods:**

The magnetic resonance imaging (MRI) examination of the patients who were sent to our clinic for investigation of neck pain between 2019 and 2020 years were evaluated retrospectively. 143 Turkish women patients between 30 and 40 ages were examined in the study. The presence and degree of IVDD was evaluated for each patient. The areas of the cervical flexor and extensor paravertebral muscles were measured.

**Results:**

No cervical disc degeneration was present in 44 (30.76%) patients (grade 1). The cervical intervertebral disc degeneration was grade 2 in 28 (19.58%), grade-3 in 41 (28.67%), and grade 4 in 30 (20.97%) patients. In early stage degeneration (grade 2), an increase was observed in the area of all cervical paravertebral flexor and extensor muscles examined. As the degree of degeneration increased (grades 3 and 4), a decrease was observed in the areas of all muscles. Statistical significance was found for musculus (m) sternocleidomastoideus, m. levator scapulae, m. splenius capitis, m. semispinalis capitis, and m. multifidus muscles (*P* = .009, *r* = −0.261; *P* = .014, *r* = −0.248; *P* = .008, *r* = −0.267; *P* = .002, *r* = −0.307; *P* = .028, *r* = −0.222, respectively).

**Conclusions:**

IVDD is common in middle-aged females with neck pain. An increase in muscles areas is observed in the early stages of cervical disc degeneration but progressive decrease develops in all cervical paraspinal muscles areas as the degree of disc degeneration increases.

## 1. Introduction

Cervical intervertebral disc degeneration (IVDD) is the main cause of spinal disorders leading to neck pain and numbness in the upper extremity.^[1]^ The intervertebral discs undergo morphological and cellular changes with age.^[2,3]^

Early diagnosis and intervention are clinically important as late stage IVDD may require surgical treatment and result in a severe financial burden by progressing to disc herniation and a narrow cervical spinal canal.

IVDD’s relationship with obesity, genetic factors, trauma, smoking, the sagittal morphology of the vertebral column, age, gender, and ethnic group has previously been investigated.^[4–14]^ Various studies have revealed a direct correlation between the degree of IVDD and the mechanical load on the spinal column.^[10]^

As far as we know, there is no study investigating the relationship between the muscle areas in the cervical region and the presence and degree of cervical IVDD.

Magnetic resonance imaging (MRI) is considered to be the best noninvasive imaging method to investigate IVDD and the paraspinal muscles in humans and animal models as it clearly shows the structural integrity of the intervertebral discs and paraspinal muscles.^[15,16]^

The aim of this study was to investigate the presence and degree of cervical IVDD, which is a part of the cervical spondylosis process, and its relationship with the cervical region muscle area.

## 2. Material and Methods

A total of 1760 patients who underwent cervical MRI at our clinic to investigate the cause of neck pain during 2019 and 2020 years were retrospectively evaluated.

A study group was selected from 143 Turkish female patients, 30 to 40 years of age, in order to eliminate age, gender and ethnic-related differences. Patients who were found on MRI to have a cervical mass, cervical congenital anomaly, cervical vertebral fracture, discitis, osteomyelitis, spondylolysis, or spondylolisthesis and patients with a symptom other than neck pain, those with a history of cervical surgery, and patients at stage 2 or above according to the Goutallier classification of muscle fatty degeneration were not included in the study to avoid any effect on the muscle area measurement results.^[17,18]^ Grade 5 degeneration of the intervertebral disc was found in very few cases (n = 2) and these patients were also not included in the study in order to prevent a statistical error. A total of 143 female patients with a mean age of 34.7 ± 0.22 years who met the inclusion criteria were finally investigated within the scope of the study. This study was approved by Alanya Alaaddin Keykubat University Medical Ethics Committee (No. E-11-04), Turkey.

All examinations were conducted with a 1.5-tesla, 16-coil MRI device (Signa: GE Medical Systems, Milwaukee, WI). Sagittal T1-weighted images (WI), sagittal T2-WI, and axial fast spin-echo T2-WI were obtained with the imaging protocol (T1WI: TR/TE 520/12 ms, echo train length 4; T2WI: 5000/102 ms, echo train length 16; slice thickness 5 mm, no slice gap, field of view 24 cm for sagittal images and 16 cm for axial images; matrix 256 × 192; 4 excitations).

All measurements and evaluations were performed with the consensus of two radiologists experienced on evaluating the musculoskeletal system.

**Figure 1.**
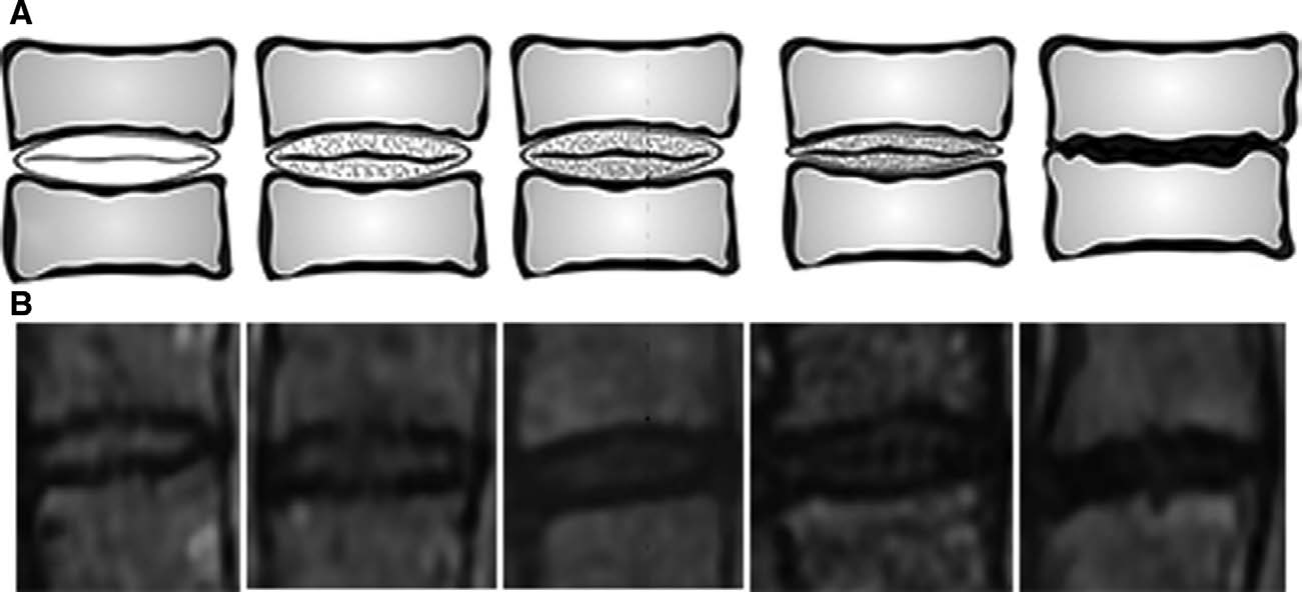
The illustration (A) and MR images (B) of the degree of cervical intervertebral disc degeneration.

The presence and degree of cervical IVDD were investigated. The evaluation was performed on sagittal T2-WI. The degree of cervical IVDD was classified in accordance with the grading system identified by Miyazaki et al (Table 1) (Fig. 1A and B).^[19]^ The patients were grouped according to the highest degree of degeneration in the intervertebral discs.

**Table 1:** Grading system for cervical intervertebral disc degeneration.

Grade	Nucleus signal intensity and nucleus structure	Distinction of nucleus and annulus	Disc height
1	Hyperintense homogenous, white	Clear	Normal
2	Hyperintense inhomogenous with horizontal band, white	Clear	Normal
3	Intermediate inhomogenous, gray to black	Unclear	Normal to decreased
4	Hypointense inhomogenous, gray to black	Lost	Normal to decreased
5	Hypointense inhomogenous, gray to black	Lost	Collapsed

The C4–C5 intervertebral disc area was identified as the reference region for the measurement of the flexor and extensor muscles in the cervical region. The C4–C5 level was chosen in order to prevent measurement errors as the axial image passing through the intervertebral disc level could be at various angles with the ground plane at other levels.

**Figure 2.**
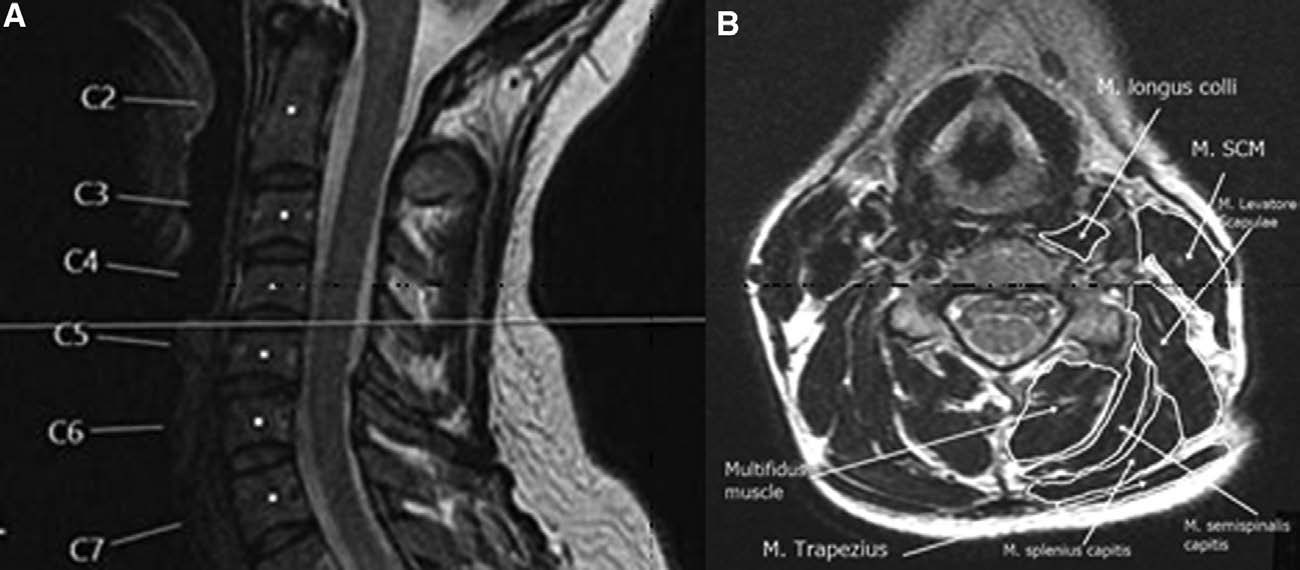
(A) Cervical mid-sagittal T2-WI. (B) Measurements of the cervical paraspinal muscles areas on axial T2-WI passing through the C4–5 intervertebral disc level.

The areas of the flexor [musculus (m) sternocleidomastoideus and m. longus colli] and extensor [m. trapezius, m. levator scapulae, m. splenius capitis (including m. splenius cervicis), m. semispinalis capitis, and m. multifidus (including m. semispinalis cervicis)] muscles were measured from both sides in axial T2-WI passing through the C4–C5 intervertebral disc level and recorded in mm^2^ by using the Sectra software program (Sectra workstation IDS7, Linköping, Sweden) (Fig. 2A and B). Muscles were differentiated with reference to an MRI anatomical atlas outlining the muscles at each level.^[20]^ Cross-sectional muscle areas was measured by manually tracing the fascial boundary of selected neck muscles (Fig. 2A and B). The muscle areas may differ according to the body fat ratio. In order to eliminate this difference and provide a standardized muscle area, these areas were divided into the area of the C4 vertebral body. Similar to the other studies in the literature, the areas of the splenius capitis and splenius cervicis muscles, and those of the multifidus muscle and semispinalis cervicis muscles were combined during the measurement as they could not be clearly differentiated.^[21]^

### 2.1. Statistical analysis

The statistical analyses of the data were performed with the SPSS software program (Version 22.0, SPSS Inc., Chicago, IL). Descriptive statistics were presented as mean ± standard deviation. The Mann–Whitney U test was used in the comparison of the muscle areas of patients with a mild degree of degeneration (grade 2) and those without degeneration (grade I). The correlation between the degeneration degree and the muscle area was investigated with Spearman’s correlation test. The *P* < .05 level was considered statistically significant.

## 3. Results

No cervical disc degeneration was present in 44 (30.76%) of the 143 patients included in the study (grade 1). The cervical IVDD was grade 2 in 28 (19.58%), grade 3 in 41 (28.67%), and grade 4 in 30 (20.97%) patients.

In early stage degeneration (grade 2), an increased was observed in the area of all cervical paravertebral flexor and extensor muscles examined (Table 2).

**Table 2:** Relationship between cervical intervertebral disc degeneration and cervical muscle areas.

Muscles	Grade 1 degeneration	Grade 2 degeneration	Grade 3 degeneration	Grade 4 degeneration	*P* value
M. sternocleidomastoideus	1.19 ± 0.04	1.25 ± 0.04	1.13 ± 0.03	1.08 ± 0.04	.009**
M. Trapezius	0.36 ± 0.02	0.37 ± 0.03	0.35 ± 0.02	0.34 ± 0.02	.467
M. levator scapulae	0.93 ± 0.04	0.97 ± 0.05	0.94 ± 0.02	0.84 ± 0.04	.014**
M. splenius capitis	0.64 ± 0.02	0.67 ± 0.03	0.60 ± 0.02	0.56 ± 0.03	.008**
M. semispinalis capitis	0.75 ± 0.03	0.8 ± 0.04	0.73 ± 0.03	0.64 ± 0.03	.002**
M. multifidus	0.86 ± 0.03	0.91 ± 0.04	0.87 ± 0.02	0.82 ± 0.03	.028**
M. longus colli	0.25 ± 0.02	0.025 ± 0.02	0.26 ± 0.02	0.023 ± 0.03	.443

**Statistically significant at *P* ≤ 0.05.

M = musculus.

A decrease in the areas of the m. sternocleidomastoideus, m. levator scapulae, m. splenius capitis, m. semispinalis capitis, and m. multifidus muscles was observed as the degeneration degree increased (grades 3 and 4) (*P* = .009, *r* = −0.261; *P* = .014, *r* = −0.248; *P* = .008, *r* = −0.267; *P* = .002, *r* = −0.307; *P* = .028, *r* = −0.222, respectively). However, no significant difference was found for the m. trapezius and m. longus colli muscles despite the presence of an inverse relationship between the degeneration degree and the muscle area (*P* = .467, *r* = −0.149; *P* = .443, *r* = −0.151, respectively) (Table 2).

## 4. Discussion

This is the first study, to the best our knowledge, to investigate the relationship between the presence and degree of cervical IVDD and the cervical muscle cross sectional areas.

The relationship of cervical degenerative changes with age has been investigated in a large number of studies. The incidence of cervical degenerative changes has been reported to increase with age by Matsumoto et al^[22]^ in their study evaluating the cervical MRI images of 497 individuals.

Cervical degenerative changes may also show gender-related differences. Wang et al^[23]^ have reported cervical degenerative changes to be more common in females.

Thus, in order to avoid probable age, gender and ethnic-related differences, the present study was performed with individuals of a specific age group, same gender, and ethnic group.

An increased IVDD degree promotes a loss of disc height and the development of annular tears and herniation.^[24]^ These structural changes are irreversible due to the limited healing potential of adult intervertebral discs.^[25]^ It is therefore extremely important to recognize all the factors associated with increased IVDD.

Various studies have investigated the relationship between IVDD in the lumbar spine and paravertebral muscle area. Miki et al^[26]^ have found a negative correlation between disc degeneration and the areas of the multifidus and erector spina muscles in a recent MRI study on 52 patients. Other studies have produced similar results.^[27]^ The current study is the first to investigate this relationship for the cervical spine and the results are consistent with the literature. Interestingly, an increase was found in the areas of the all cervical paraspinal muscles in the presence of early stage (grade 2) degeneration in this study. Our results have revealed that the muscles areas increased in the early stages of spinal loading (grade 2), but progressive decrease in muscle areas developed in the later stages of IVDD (grades 3 and 4).

A relationship has previously been reported between paravertebral muscle degeneration and chronic spinal pain, degenerative disc disorder, radiculopathy, and scoliosis.^[17,28,29]^ The paraspinal muscles support the spinal column and help decrease compressive forces on the disc.^[30]^ Weakened paraspinal muscles result in more rapid development of degenerative spinal pathologies. Therefore planning rehabilitation programs by taking into account the fact that the clinical picture may be accompanied by a decrease in muscle areas will be beneficial especially for patients with grade 3 and higher disc degeneration.

Our study had a couple of limitations. The first is that we did not query the history of smoking, alcohol use, obesity, occupation, and chronic diseases such as diabetes that may be associated with IVDD.^[31]^ The second limitation is that the evaluators being aware of the degree of IVDD during the evaluation could lead to errors, although unlikely.

## 5. Conclusion

IVDD is common in middle-aged females with neck pain. An increase in cross sectional areas of all cervical paraspinal muscles is observed in the early stages of cervical disc degeneration but progressive decrease develops in all cervical paraspinal muscles as the disc degeneration increases.

## Author contributions

**Conceptualization**: Y.Y. and T.E.

**Data curation**: Y.Y., T.E., and E.T.

**Formal analysis**: T.E. and Y.Y.

**Investigation**: Y.Y. and E.T.

**Methodology**: Y.Y.

**Resources**: Y.Y., T.E., and E.T.

**Software**: Y.Y.

**Supervision**: Y.Y.

**Writing - original draft**: Y.Y. and T.E.

**Writing - review & editing**: Y.Y., T.E., and E.T.

**Conceptualization:** Tarkan Ergun, Yavuz Yuksel.

**Data curation:** Tarkan Ergun, Yavuz Yuksel.

**Formal analysis:** Tarkan Ergun, Yavuz Yuksel.

**Methodology:** Ebru Torun, Tarkan Ergun, Yavuz Yuksel.

**Resources:** Yavuz Yuksel.

**Supervision:** Ebru Torun, Yavuz Yuksel.

**Visualization:** Yavuz Yuksel.

**Writing – original draft:** Ebru Torun, Tarkan Ergun, Yavuz Yuksel.

**Writing – review & editing:** Yavuz Yuksel.

## References

[1] Schoenfeld AJ Nelson JH Burks R. Incidence and risk factors for lumbar degenerative disc disease in the United States military 1999-2008. Mil Med. 2011;176:1320–4. 22165663 10.7205/milmed-d-11-00061

[2] Kjaer P Leboeuf-Yde C Korsholm L. Magnetic resonance imaging and low back pain in adults: a diagnostic imaging study of 40-year-old men and women. Spine. 2005;30:1173–80. 15897832 10.1097/01.brs.0000162396.97739.76

[3] Haefeli M Kalberer F Saegesser D. The course of macroscopic degeneration in the human lumbar intervertebral disc. Spine. 2006;31:1522–31. 16778683 10.1097/01.brs.0000222032.52336.8e

[4] Liuke M Solovieva S Lamminen A. Disc degeneration of the lumbar spine in relation to overweight. Int J Obes (Lond). 2005;29:903–8. 15917859 10.1038/sj.ijo.0802974

[5] Battié MC Videman T Gibbons LE. Volvo award in clinical sciences. Determinants of lumbar disc degeneration. A study relating lifetime exposures and magnetic resonance imaging findings in identical twins. Spine. 1995;1995:2601–12. 8747238

[6] Livshits G Popham M Malkin I. Lumbar disc degeneration and genetic factors are the main risk factors for low back pain in women: the UK twin spine study. Ann Rheum Dis. 2011;70:1740–5. 21646416 10.1136/ard.2010.137836 PMC3171106

[7] Sambrook PN MacGregor AJ Spector TD. Genetic influences on cervical and lumbar disc degeneration: a magnetic resonance imaging study in twins. Arthritis Rheum. 1999;42:366–72. 10025932 10.1002/1529-0131(199902)42:2<366::AID-ANR20>3.0.CO;2-6

[8] Carragee EJ Don AS Hurwitz EL. 2009 ISSLS prize winner: does discography cause accelerated progression of degeneration changes in the lumbar disc: a ten-year matched cohort study. Spine. 2009;34:2338–45. 19755936 10.1097/BRS.0b013e3181ab5432

[9] Battié MC Videman T Gill K. 1991 Volvo award in clinical sciences. Smoking and lumbar intervertebral disc degeneration: an MRI study of identical twins. Spine. 1991;16:1015–21. 1948392

[10] Ergün T Lakadamyali H Sahin MS. The relation between sagittal morphology of the lumbosacral spine and the degree of lumbar intervertebral disc degeneration. Acta Orthop Traumatol Turc. 2010;44:293–9. 21252606 10.3944/AOTT.2010.2375

[11] Weiler C Schietzsch M Kirchner T. Age-related changes in human cervical, thoracal and lumbar intervertebral disc exhibit a strong intra-individual correlation. Eur Spine J. 2012;810:818. 10.1007/s00586-011-1922-3 PMC3535216 21837413

[12] Boos N Weissbach S Rohrbach H. Classification of age-related changes in lumbar intervertebral discs: 2002 Volvo Award in basic science. Spine (Phila Pa 1976). 2002;27:2631–44. 12461389 10.1097/00007632-200212010-00002

[13] Siemionow K An H Masuda K. The effects of age, sex, ethnicity, and spinal level on the rate of intervertebral disc degeneration: a review of 1712 intervertebral discs. Spine (Phila Pa 1976). 2011;36:1333–9. 21217432 10.1097/BRS.0b013e3181f2a177 PMC3117081

[14] Niu G Yang J Wang R. MR imaging assessment of lumbar intervertebral disk degeneration and age-related changes: apparent diffusion coefficient versus T2 quantitation. AJNR Am J Neuroradiol. 2011;32:1617–23. 21799044 10.3174/ajnr.A2556 PMC7965379

[15] Modic MT Ross JS. Lumbar degenerative disk disease. Radiology. 2007;245:43–61. 17885180 10.1148/radiol.2451051706

[16] Waris E Eskelin M Hermunen H. Disc degeneration in low back pain: a 17-year follow-up study using magnetic resonance imaging. Spine (Phila Pa 1976). 2007;32:681–4. 17413474 10.1097/01.brs.0000257523.38337.96

[17] Mengiardi B Schmid MR Boos N. Fat content of lumbar paraspinal muscles in patients with chronic low back pain and in asymptomatic volunteers: quantification with MR spectroscopy. Radiology. 2006;240:786–92. 16926328 10.1148/radiol.2403050820

[18] Battaglia PJ Maeda Y Welk A. Reliability of the Goutallier classification in quantifying muscle fatty degeneration in the lumbar multifidus using magnetic resonance imaging. J Manipulative Physiol Ther. 2014;37:190–7. 24630770 10.1016/j.jmpt.2013.12.010

[19] Miyazaki M Hong SW Yoon SH. Reliability of a magnetic resonance imaging-based grading system for cervical intervertebral disc degeneration. J Spinal Disord Tech. 2008;21:288–92. 18525490 10.1097/BSD.0b013e31813c0e59

[20] Au J Perriman DM Pickering MR. Magnetic resonance imaging atlas of the cervical spine musculature. Clin Anat. 2016;29:643–59. 27106787 10.1002/ca.22731

[21] Snodgrass SJ Croker C Yerrapothu M. Cervical muscle volume in individuals with idiopathic neck pain compared to asymptomatic controls: a cross-sectional magnetic resonance imaging study. Musculoskelet Sci Pract. 2019;44:102050. 31451399 10.1016/j.msksp.2019.102050

[22] Matsumoto M Fujimura Y Suzuki N. MRI of cervical intervertebral discs in asymptomatic subjects. J Bone Joint Surg Br. 1998;80:19–24. 9460946 10.1302/0301-620x.80b1.7929

[23] Wang XR Kwok TCY Griffith JF. Prevalence of cervical spine degenerative changes in elderly population and its weak association with aging, neck pain, and osteoporosis. Ann Transl Med. 2019;7:486. 31700922 10.21037/atm.2019.07.80 PMC6803181

[24] van Goethem JWM van den Hauwe L Parizel PM. Spinal imaging: diagnostic imaging of the spine and spinal cord. ed 1. Berlin: Springer. 2007

[25] Adams MA Roughley PJ. What is intervertebral disc degeneration, and what causes it? Spine (Phila Pa 1976). 2006;31:2151–61. 16915105 10.1097/01.brs.0000231761.73859.2c

[26] Miki T Naoki F Takashima H. Associations between paraspinal muscle morphology, disc degeneration, and clinical features in patients with lumbar spinal stenosis. Prog Rehabil Med. 2020;5:20200015. 32844128 10.2490/prm.20200015 PMC7429555

[27] Sun D Liu P Cheng J. Correlation between intervertebral disc degeneration, paraspinal muscle atrophy, and lumbar facet joints degeneration in patients with lumbar disc herniation. BMC Musculoskelet Disord. 2017;18:167. 28427393 10.1186/s12891-017-1522-4 PMC5399427

[28] Freeman MD Woodham MA Woodham AW. The role of the lumbar multifidus in chronic low back pain: a review. PM R. 2010;2:142–6; quiz 1 p following 167. 20193941 10.1016/j.pmrj.2009.11.006

[29] Lee JC Cha JG Kim Y. Quantitative analysis of back muscle degeneration in the patients with the degenerative lumbar flat back using a digital image analysis: comparison with the normal controls. Spine (Phila Pa 1976). 2008;33:318–25. 18303466 10.1097/BRS.0b013e318162458f

[30] Kader DF Wardlaw D Smith FW. Correlation between the MRI changes in the lumbar multifidus muscles and leg pain. Clin Radiol. 2000;55:145–9. 10657162 10.1053/crad.1999.0340

[31] Teles Filho RV Abe GM. Genetic influence in disc degeneration - systematic review of literature. Rev Bras Ortop. 2020;55:131–8. 10.1055/s-0039-1692626 PMC7186076 32346187

